# The Hospital. Nursing Section

**Published:** 1904-04-02

**Authors:** 


### The Hospital.

### Nursing Section.

Contributions for this Section of "The Hospital" should be addressed to the Editor, "The Hospital" Nursing Section, 28 & 29 Southampton Street, Strand, London, W.C.

No. 914.—Vol. XXXVI. *SATURDAY, APRIL 2, 1904.*

### Notes on News from the Nursing World.

### PARLIAMENT AND REGISTRATION.

The Registration Bill of the Royal British Nurses' Association is backed by Mr. Claude Hay, Mr. Bignold, Mr. H. D. Greene, Captain Bagot, Lord Cecil Manners and Mr. Malcolm. It is rather curious that all these gentlemen are Unionists. In the case of a non-party measure it is sound policy to enlist the support of members sitting on both sides of the House of Commons. There is a movement to refer both this Bill and that in charge of Dr. Farquharson to a Select Committee. We see no objection to this course. It cannot be pretended that the question is urgent, and the report of a Select Committee, even when it is issued, is not necessarily the precursor of legislation.

### MR. JOHN LANGTON ON THE NURSING VOCATION.

The annual meeting of the Shoreditch and Bethnal Green District Nursing Association was held last week at the residence of Sir John and Lady Lawrance in Onslow Square, and there was an excellent attendance. The principal speaker was Mr. John Langton, F.R.C.S., who paid a tribute to the nurses of the United Kingdom, whom he calculated had reached a total of eighty thousand. He maintained that at present the work of a nurse should be called a vocation rather than a profession, though he believed that in the years to come the latter would be the correct designation. He confessed that he should like to see even Jubilee nurses trained in hospitals for three years. A nurse's life was a hard one, wherever she laboured, but he thought that the nurse who had to tend the sick poor in their own homes had more onerous duties because she had neither sister nor matron always at hand to consult. Morally also much responsibility rested on a district nurse, for in any case, she was on the constant trail of sorrow, and it depended largely upon her how sorrow left her patient. Alluding to "Florence Nightingale," Mr. Langton said he used the name of this illustrious Englishwoman, the founder of trained nursing, without a prefix because the simple name brought closer to all the woman who had always been the friend and companion of those in trouble. Other speakers included the Rev. H. P. Cronshaw, the Rev. H. V. Eck, and the Rev. William Cuff. Mr. Cronshaw's graphic description of his helplessness during the illness last year of the Bishop of London, whose domestic chaplain he was at the time, until he obtained a nurse, excited great interest.

### RAISING THE STANDARD FOR MENTAL NURSES.

The system of the Medico-Psychological Association is impeached by Miss Laura Evans, formerly head nurse at Berrywood Asylum, Northampton. She maintains that a mental nurse should have at least three years' training in an asylum, attend lectures and pass examinations the same as in a general hospital, before she is certified as a qualified nurse. This, she points out, was done at Berrywood, and Miss Evans, in support of her contention, says that nurses trained at Berrywood have superseded those trained in a general hospital, in good positions. She also affirms that several girls who failed completely in their examination at Berrywood got the Medico-Psychological Association certificate a few months after entering another asylum, although they must be a year at Berrywood before they can pass the first examination. Our view is that the system of the Medico-Psychological Association is capable of improvement, and that Miss Evans, in advocating the elevation of the standard, is serving the best interests of mental nurses.

### THE ENROLMENT OF CERTIFIED MIDWIVES.

A meeting of the Central Midwives Board was held on Thursday, last week. On consideration of applications for approval of certificate as a qualification under Section 2 of the Act, the certificates of the National Maternity Hospital, Dublin, Birmingham Workhouse Infirmary, Bristol Royal Infirmary, Brownlow Hill Workhouse Hospital, Liverpool, Essex County Cottage Nursing Society, Gloucester District Nursing Society, Hull Lying-in Charity, and Ipswich Nurses' Home were approved for the training of midwives under Section C of the rules, subject to an undertaking to comply with the requirements of Section E of the rules. The following registered medical practitioners were approved as teachers under Section C 1 (3) of the rules: A. B. Batley, M.R.C.S., A. B. Calder, M.R.C.S., John Gutch, M.D., E. S. Hoare, M.R.C.S., Wilfrid Kingdon, M.B., E. J. Maclean, M.D., Thomas Pimley, M.B., Penrose L. W. Williams, M.R.C.S.; and Eleanor Heatley, E. S. Sibery, E. S. Tate and Bessie M. Worrall were approved as certified midwives under Section C 1. (2), for the purpose of signing certificates of attendance (Forms III. and IV.). After the consideration of applications for certificates the names of 849 women were passed under Section 2 of the Act, and ordered for entry on the roll. The total number of midwives at present entered on the roll is 3,415, and they are thus qualified:—Royal College of Physicians of Ireland, 1; Obstetrical Society of London, 953; Rotunda Hospital, Dublin, 43; Coombe Hospital, Dublin, 12; Queen Charlotte's Hospital, 52; Liverpool Lying-in Hospital, 15; British Lying-in Hospital, 2; Glasgow Maternity Hospital, 32; St. Mary's Hospital, Manchester, 53; City of London Lying-in Hospital, 6; Royal Maternity Hospital, Edinburgh, 5; Salvation Army Maternity Hospital, 3; and Women in *boná-fide* practice July 1904, 2,238.

### THE TRAINING OF THE OPHTHALMIC NURSE.

The particulars of the training at the Royal Ophthalmic Hospital given by the matron to our commissioner, which appear in another column, will be read with special interest by those who desire to begin nursing at an early age. The advantages of being able to nurse ophthalmic cases are indisputable, and two years of training at the Royal Ophthalmic Hospital can scarcely fail to be regarded as an excellent preparation for a general hospital. The probationers in that institution have also opportunities of gaining experience in ordinary cases, and the sisters under whom they work are all fully trained hospital nurses.

### DISTRICT NURSING AS AN INVESTMENT.

The financial position of the Bristol District Nurses' Society is still by no means satisfactory. At the annual meeting it transpired that the deficit for 1903 exceeds £56, although Mr. George White guarantees £70 for the year's expense of a nurse, and another generous friend provides the entire annual expense of the nurse in St. Jude's district. The Bristol Guardians also contribute a hundred guineas. Dr. Mitchell Clarke, who was one of the speakers at the meeting, said that, putting it upon commercial grounds, he believed that it would pay a large city like Bristol to maintain a nurse for every parish. This is an aspect of district nursing which merits the attention of persons who, though not in the habit of supporting charities from benevolent motives, may be willing to help them if they think that by doing so they can in the long run save their own pockets.

### HOW NURSES ARE MANUFACTURED IN FRANCE.

With reference to the translation which we published a few weeks ago of an article entitled "Journal d'une Elève," in *La Revue des Deux Mondes*, a communication purporting to be a reply has since appeared in *Le Progrès Medical*, signed "Julien" Noir. But beyond alluding to the well-known fact that Dr. Bourneville, physician to the Bicêtre, has for a long time been doing his best to remedy a disgraceful state of affairs, and in consequence incurred much odium, it consists almost entirely of *badinage*. The writer, alluding to the instruction on the use of the douche, in which the subjects were idiot children, affirms that they are accustomed to it, that it does them good, and that the sight of the afflicted children should have aroused compassion and sympathy instead of a false modesty. Most of the points dealt with are of minor, not major, importance; and we do not think that the main statements are seriously impugned.

### MIDWIFERY SCHOLARSHIPS.

The Incorporated Midwives' Institute are offering scholarships for free or assisted training in midwifery. Applications can be sent in to the Secretary of the Institute, 12 Buckingham Street, Strand. As the Institute has done excellent work in the cause of the education of midwives, it should have the choice of the best possible material in the selection of candidates. We hope that by means of these scholarships much may be done in the future to encourage the supply of well-trained women of the right stamp throughout the country.

### BOROUGH COUNCILS AND DISTRICT NURSING.

At the annual meeting of the Central St. Pancras District Nursing Association, Sir William Collins testified from personal experience to the good work done by the nurses. He was glad that the importance of proper training was beginning to be realised, for however true it might be that women were ministering angels, he must always protest against the idea that every woman was a born nurse. Sir William, commenting on the approval expressed by Lady Jeune of the suggestion that public bodies, such as the London County Council, should financially assist voluntary bodies like the Queen's Nurses, said he believed that the London County Council had no power to render the assistance proposed, but Borough Councils had. There are cases in which it might be desirable for Borough Councils to exercise the power, but the more District Nursing Associations depend for revenue upon the contributions of regular subscribers the better. The danger of receiving grants in aid from public bodies is that there should be a corresponding slackening of voluntary support.

### NURSES' UNION.

The annual meeting of the Nurses' Union took place on Thursday of last week. At 3 o'clock a short service was held at Holy Trinity, Marylebone, and the Rev. E. Grose Hodge gave an address. A reception by Miss Dashwood, hon. secretary of the Nurses' Union afterwards took place at her residence, 5 Cambridge Gate. In the drawing-room vocal and instrumental music and recitations went on from 3.45 to 8 p.m., and the Rev. Canon Girdlestone made a short speech. The company included nurses from all parts, and Miss Dashwood, her sisters and friends were untiring in their efforts to afford their guests enjoyment.

### A CRISIS AT KNOTTINGLEY.

We regret to learn that a crisis has arisen in the affairs of the Knottingley District Nursing Association. Established in February 1895, with Lady Gwendolen Ramsden as president, and a committee consisting of four other ladies and the two doctors in the town, the Association was carried on with much success and without friction until the beginning of last year. Then, it appears, the medical men on the committee proposed that a rule which provides that the nurse shall work under them and apply to them at once in any case of difficulty, should be altered so as to read, "Only such cases will be undertaken as are under the care of a medical man." The majority of the committee, however, declined to assent to the change, and the medical men subsequently retired from the Association. Their action has now been followed by an announcement from Lady Gwendolen Ramsden and her colleagues that they cannot continue the work, and will therefore resign on April 18th. In the circular they have sent out they state that "any balance that remains of the present year's subscriptions after paying the current expenses will be returned to the subscribers," from which it is evident that unless prompt action be taken the organisation will come to an untimely end. This would be a great pity, as both the well-to-do and the poor of Knottingley are unanimous in their appreciation of the value of the work done by the district nurse. On the surface, the difficulty which threatens to involve dissolution ought not to be insuperable.

### A MIDWIFE SENT TO PENAL SERVITUDE.

Last week at the Central Criminal Court Bertha Baudach, a qualified midwife and a native of Germany, was convicted of having caused the death of a young married woman by an illegal operation, and sentenced by Mr. Justice Grantham to seven years' penal servitude. The sentence is terrible, but as the prisoner had been convicted of another shocking offence in 1895 and served five years' penal servitude for it, we cannot say that it was unduly severe. It is remarkable that, after being five years in penal servitude, Baudach should apparently have been able to resume her calling as a midwife. This is the sort of scandal that will hardly be possible under the provisions of the Midwives Act.

### PLYMOUTH GUARDIANS AND THEIR CHARGE NURSE.

For two successive weeks the Plymouth Guardians elected a charge nurse, only to find at their next meeting that they had to go over the ground again. The first nurse appointed was shown over the House and declined the post. The second, who followed her example, wrote informing the Board that she thought a charge nurse should see the Infirmary and accommodation before accepting such a responsible position. It will probably not be disputed that the Infirmary building is scarcely up to date, but as the Guardians have done a good deal to render the work of the nurses as little irksome as possible, it is discouraging to them that two nurses should decline to take up their duties immediately after appointment. The Hospital Committee have been directed to inquire into the circumstances of the refusals, and meanwhile the Guardians have made a third appointment.

### PROGRESS AT DEAL.

One of the features of the annual meeting of the Deal and Walmer Victoria Hospital was that the speakers were able to refer in terms of satisfaction to the progress of the private nursing institution. There was a time when it was supposed that the hospital itself was not necessary, but this impression has been entirely dissipated by its record of service; and its report of the work of the private nursing institution, which shows that the nurses earned nearly £200 and could have done better if the staff had been larger, proves that this was a very real want in the town. The present need is that the nurses, instead of being housed some distance from the hospital, should be close at hand, as on more than one occasion the matron of the hospital has had to go in the middle of the night to a nurse's lodging in order to send her to an urgent case. This, however, is about to be remedied, the Committee having very wisely decided to take an opportunity afforded them of purchasing the house adjoining the hospital. Last year there was a gain on the nursing branch of about £50.

### NO TRAINED NURSE NEED APPLY.

At a meeting of the Carnarvon Guardians, the report of the Visiting Committee also recommended that the Board should advertise for a trained nurse in place of a nurse who had resigned, was considered. Mr. H. Parry said that he failed to see why they should appoint a trained nurse, "who might be entitled to be called so after an examination of perhaps ten minutes." It is satisfactory to learn that this remark, which was silly if it was not ignorant, did not pass unnoticed, and that another speaker pointed out that a trained nurse has not only to pass an examination, but also to serve in a hospital for some years. However, the Carnarvon Guardians, having listened to a dissertation from another member upon the importance of not placing a younger person above an untrained nurse already in the workhouse, "just because the former happened to be trained," declined to have anything to do with a trained nurse, and decided to advertise "simply for a nurse."

### BICYCLES WANTED IN NORTH LONDON.

At the annual meeting of the North London Nursing Association it was stated that the nurses were badly in want of some more bicycles. They already possess two, which have proved of immense value in enabling them to go about quickly over the long distances they have to traverse in their large districts; but as 1,402 patients were tended and 33,634 visits paid during 1903, it will be realised that two are insufficient. We are sorry to notice an adverse balance of £47 in the treasurer's statement; and though a society which has been in existence for 26 years is used to ups and downs, it may be hoped that next year the income will, at any rate, be sufficient to meet the expenditure.

### SHORT ITEMS.

A special service was held in the chapel at St. Mary's Hospital, Paddington, on Friday, many nurses being present. The preacher was the Archdeacon of Middlesex, who dwelt on the necessity of rest in work.—The Peterhead Nursing Association which has just been formed is, we understand, affiliated with the Scottish branch of Queen Victoria's Jubilee Institute.

### The Nursing Outlook.

"From magnanimity, all fear above;
From nobler recompense, above applause,
Which owes to man's short outlook all its charm."

### NURSING IN GREAT BRITAIN.

### IV.—The Nurse-training Schools.

The great nurse-training schools, especially in London, may, from their point of view, be well content with their position and the work they are accomplishing, largely in their individual interests and indirectly for the public at large. The London Hospital, for instance, in the year 1892, received £4,211 from its private nursing institution. That sum presumably represents the profit made by this single hospital out of the earnings of the nurses which it supplied to private families and others in a single year. However satisfactory such a state of things may be to the hospital authorities, it cannot be entirely just to the nurses who earn the money, or to the public who employ them, unless the London Hospital provides, as Guy's Hospital provides, under a definite scheme, which inalienably secures to the women workers they employ, a liberal provision in the day of sickness and a handsome pension—say at 50 years of age. These remarks apply equally to every other nurse-training school which has a private staff of nurses and makes a profit out of their work without securing the individual worker against the day of necessity. It is manifestly undesirable for public institutions to farm nurses at all, unless they devote the profits realised on the business, in the first instance, to promote the welfare, safety and comfort of their nursing staff. Another point which requires elucidation is to what extent, if at all, any nurses' training-school sends out to private families and cases nurses who have not completed their training and are not in possession of a three years' certificate.

The first point just dealt with is more or less of a domestic character, but it has an important bearing upon private nursing, because every man or woman who pays a handsome weekly sum when ill for a trained nurse has the right to expect that that nurse will receive practically the whole of what she may earn. Our immediate concern is, however, to ascertain how far the great nurse-training schools discharge the wider duties which they owe to the whole body of nurses. This aspect of the question has received far too little attention in the past from the authorities of the nurse-training schools. As Lady Helen Munro-Ferguson recently pointed out, not only each nurse-training school, but practically every hospital, whatever its size and standing, is at present a self-constituted training school. Each works as a separate unit, carries out its own theories of training, and confers its own certificate. This is the origin of most of the confusion which exists in the nursing world in England to-day. It accounts for the difficulty of securing the services of a fully-qualified trained nurse when she is required by a private family. Illness comes suddenly, and those members of the public who adopt the recommendation of the individual nurse-training schools to apply to them for a nurse necessarily find that no nurse is available at the moment, because in practice it is impossible for any individual school to maintain a sufficiently large staff of private nurses to meet the increasing popular demand. Yet, in their memorial, those of the nurse-training schools who are opposing registration, claim that they provide, under this system, everything that the public can reasonably ask for. They, further, point out—and correctly—that character in a nurse is more important than the ability to pass the most difficult of examinations; yet they claim full recognition for their certificate of training, because they insist upon the importance of examination tests. Until each nurse-training school maintains a sufficient number of nurses on its private staff to meet in every real sense the requirements of the public—and we have shown they can practically never hope to do this—the present attitude of a large number of the training schools is illogical and contrary to their best interests. It is further true that the whole conception of the private staff is frequently selfish from a hospital point of view, for it farms nurses out at a profit, keeps that profit, and does not expend it, as ought to be done, for the benefit of the nurses whose work has produced the revenue in question.

Again, in matters of education the majority of the nurse-training schools have refused to use their influence to secure a uniform standard amongst themselves, or to promote an organisation which would protect the public and the highly qualified nurse by securing a uniform examination and certificate of general efficiency in professional training. Such a standard would remove most of the difficulties and confusion which at present paralyse the profession of nursing in this country. The institution of these reforms without delay is urgently required. They would safeguard the interests of the public, the nurses and the schools. They would secure that the present demand for registration should be dealt with on its merits. Thus the risk of registration being forced through Parliament as a panacea, whilst it must merely prove in practice a failure as matters are at present, would be effectually removed.

It is patent to those who are most familiar with public and hospital opinion that the nurse-training schools must bestir themselves. They cannot continue to maintain their ostrich-like attitude, for the English are a practical nation, and they will not much longer suffer the present deadlock, which is mainly due to the failure of those immediately responsible to discharge their obligations by voicing opinion and setting themselves at the head of a movement to create order out of the existing chaos.

### The Modern Nursing of Consumption.

By Dr. Jane H. Walker, Physician to the New Hospital for Women, London, and Medical Superintendant of the East Anglian Sanatorium, Nayland, Suffolk.

### LECTURE II.

### Symptoms of Phthisis.

After long exposure to the disease, or after some debilitating illness, such as typhoid, influenza, or perhaps with no definite antecedent cause, a patient may complain of want of appetite, tiredness, and lassitude, and following these, more or less rapid loss of flesh. These three symptoms are not special to phthisis, they may occur after any illness, or from various causes. They may have no special significance, or they may very readily prepare the soil upon which the tubercle baccili are capable of planting themselves, or they may be due to the toxins secreted by the already flourishing bacilli. Be this as it may, these early symptoms are generally speedily followed by others more special to the disease. These are :—

1. *Cough.*—This is often the first thing of which a patient complains. It is due to the irritation of the lungs, produced by the formation of the tubercles, and the consequent congestion around the nodules.

2. *Anæmia.*—Due to deficient aeration of the lungs, and to the systemic disturbance caused by the toxins.

3. *Weakness.*—Due to intensification of the causes mentioned above.

4. *Loss of appetite, sickness, and dyspepsia.* These symptoms are no doubt partly due to the bacillary toxins, but are in most cases largely caused by the custom which most people in this country still adopt, of shutting anyone with a cough in a stuffy room, feeding him on invalid messes, and giving some nauseating cough mixture, which does neither harm nor good to the tubercle bacilli.

5. *Pain.*—This may be a very early symptom of the disease, and indicates, as a rule, involvement of the pleura. The pain may disappear if effusion take place, which in its turn may be absorbed, and the attack for the time being at any rate be ended.

6. *Night Sweats.*—The cause of night sweats is by no means clear. They may be due to an accumuulation of carbonic acid in the blood—a condition produced largely by insufficient fresh air. Whatever the cause, patients in sanatoria very rarely have night sweats, although the temperature may continue after admission to go up to 102-103 degrees F. every night.

It must not be imagined that all these symptoms are complained of in every case. Sometimes no cough is present, in other cases there is no wasting. Patients with quite extensively affected lungs may look well, have good appetites, and may not have lost weight. Such cases not infrequently take longer to recover than do those in which we find wasting and whose looks correspond more nearly with the severity of their lung condition. There may be no fever, though the lungs are much involved; or there may be high temperatures, rapid pulse, and general disturbance of health far in excess of anything local which we can hear. These cases, again, often do badly; the system appears to be incapable of resisting the illness, and the general health and strength of the patient fail rapidly without any proportionate lung disease.

### Classification of Cases.

In making any classification of consumption it cannot be too strongly insisted upon that we are dealing with a general disease, the local manifestation of which is mainly situated in the lungs. It would be as untrue to count rheumatic fever as a disease of the joints as it is to call consumption a disease of the lungs only.

Some people divide consumption into three stages: the first when the bacilli are invading the tissues, and nodules with accompanying consolidation are found; the second when there is fever and more advanced tubercle formation; the third when cavities are present.

These divisions present little to one's minds; a patient may be only in the first stage and yet have fever, or he may have a cavity which is drying up, his temperature may be good, his appetite and general condition excellent; and yet, according to the classification, the possession of a cavity would place him in the third stage, a term which, besides having a death-bed knell about it, conveys no pathological or clinical picture.

Moreover, intended as these lectures are for nurses, any classification based merely upon the signs in the lungs, would be of no value; therefore it appears better to divide cases (though the division is equally arbitrary) into (*a*) acute, (*b*) subacute, (*c*) chronic.

*Acute.*—Under this heading we include those cases in which the disease rapidly advances and in which no treatment is of any avail. The disease in these cases is more systemic than of the lungs, and the patients frequently fall into the typhoid condition before death.

*Subacute.*—To this class belong far the largest number of cases; in it we should include all those cases which are prolonged over months, whether they finally do well or badly. Of course, many subacute cases tend to become chronic., especially under the open-air treatment. Cases which in the old days would have died in a few months will live for months or years, even though the disease may not be cured; such cases not infrequently delevop kidney trouble (lardaceous disease)—a comparatively rare occurrence in phthisis pulmonolis in former times.

*Chronic.*—We would group under this heading those cases of very slow consumption, in which the process shows no signs of advancing to cavity formation. The disease does not stop, but progresses gradually, more nodules being formed, these nodules tending to become hard and fibrous; such cases are often called "fibroid phthisis." They are not very common, but the term is loosely used. Probably real fibroid phthisis is never curable. We have had cases sent to us diagnosed as "fibroid" which have done very well under the open-air treatment.

### Acute Phthisis.

In these cases the patients are apparently unable to withstand the toxins secreted by the bachilli, and we have frequently also to deal with mixed infection. There is progressive emaciation, weakness, cough, pyrexia, night sweats, and in spite of the open-air or any other treatment, the patient dies rapidly, generally of asthenia, or perhaps of extensive and very quick excavation of the lungs, complicated by abdominal symptons These cases are rare in children. We have had one case of a child of four, in whom the disease presented all the symptoms generally found in older persons, and the child died within three months of the beginning of the illness, of acute phthisis pulmonalis.

The treatment of the case is practically that of any acute and severe illness, such as typhoid or scepticæmia. The patient's strength must be supported as much as possible, all exertion must be avoided; he must be washed, fed, and moved entirely by the nurse. Scrupulous cleanliness is of course required; bed-sores are always to be feared. All excreta, particularly all sputum, should be disinfected before being taken out of the patient's room. The nurse must keep a diary of the temperature, pulse, respiration, evacuations, and should note any changes in the patient's condition to report to the doctor at his visit.

Should the case be in a private house the nurse may have to give such stimulants as brandy, oxygen, strychnine, or amyl nitrate at her discretion; she should, of course, note not only the quantity given and the time at which it was administered, but also exactly what the condition was which called for the remedy.

A really observant nurse is a blessing to doctor and patient alike, and a good nurse should show herself observant not only in carrying out strictly the daily medical orders, but especially if the case be in the country, in anticipating any emergency likely to happen. In a sanatorium, of course, everything will be, or should be, at hand, but foresight and thoughtfulness on the nurse's part are specially required in nursing private patients.

### The Nurses of the Royal London Ophthalmic Hospital.

INTERVIEW WITH THE MATRON. BY OUR COMMISSIONER.

The nursing at special hospitals is, of course, on different lines to that at general hospitals, but it is not less interesting, nor less important, and it is satisfactory to find that special institutions are keeping pace in this respect with the times. It is now about twelve months ago since the Royal London Ophthalmic Hospital in City Road—which is celebrating its centenary this year—became a teaching school for nurses.

"That is to say," explained Miss B. M. Cooke, the lady superintendent, when I called to see her, "until the beginning of last year, the period of training was only for one year. Now it is two years.

### The Probationers.

"How is your nursing staff composed at the present time?" I inquired.

"There are seventeen nurses and six sisters. One of the latter is night sister, another, home and holiday sister, a third is in charge of the men's floor, which has 44 beds, a fourth in that of the women's floor, also with 44 beds, a fifth in that of a floor containing 15 children's beds and 15 beds for men; and a sixth is responsible for the out-patients' department, which is on a very large scale."

### Training and Age of Admission.

"What theoretical training is given to the probationers?"

"One of the surgeons and myself give lectures. At the end of two years there is an examination, which must be passed before a probationer obtains her certificate."

"And the age of admission?"

"Twenty-one, and up to any reasonable age. This means that a girl can have her training here before she becomes a probationer at a general hospital. I think that the two years' work must render her more valuable when she goes to a general hospital. It is certainly likely to be a considerable advantage to her after she has received her general training, and renders her eligible to be sister or matron in a general hospital."

### Staff Nurses' and Sisters' Salaries.

"Do you pay a salary at once?"

"Yes, £12 the first year, and £20 the second. We also provide indoor uniform; if the nurses like to wear uniform outside, they have to get it themselves. Of the 17 nurses, 11 are probationers, and the remainder are staff nurses, who have had training in other hospitals."

"For three years?"

"I prefer to have them with three years' training, but I take them if they have had two. The salaries are £20 to £30, the amount varying according to the period of previous training."

"The sisters, I conclude, have always had three years' training?"

"We do not take them without a three years' certificate. The salaries of the sisters commence at £35 and increase to £40. A new sister has to engage to come for a year; chopping and changing in a special hospital would be disastrous. The night sister also remains on night duty for a year."

### Night Duty.

"How many of the staff are on night duty with her?"

"There is one nurse for each floor, and a young probationer to do whatever is necessary. A night porter is always available. All our nurses go on night duty for two months at a time. Their hours are from 9 p.m. to 8.30 a.m., during which they are not allowed to leave the wards. But they have two meals in the course of the night. After they have had their dinner at 8.45 a.m., they can go out from 9.15 until 12.30. They are expected to be in bed by one, and they have breakfast at 8.30 p.m."

### Day Duty and Holidays.

"When do the day nurses go on duty?"

"At 7 a.m. They have two hours off duty every day, and half an hour for dinner and tea. I mean a full half hour, and tea is served in the dining-room. Probationers have half a day off every fortnight—from 2 to 10 p.m.—and a day every month. Both sisters and staff nurses have two hours off every day, and half a day once a week. Staff nurses have a whole day once a month, and sisters once a fortnight. If any nurse wants to go home the night before her day off, I am always willing to grant permission. It does the nurses good to get them out of the City Road for a night. As to holidays, the probationers have a fortnight in the year, the staff nurses three weeks, and the sisters a month."

### Sisters with Special Training.

"You were sister here for some years, I think, Miss Cooke?"

"For four and-a-half I was out-patient sister, and then I was appointed Matron. I was trained at the County Hospital, Taunton. I am very fond of the nursing of Opthalmic patients, and in fact prefer it to anything."

"Had you any experience of eye nursing before you came to this Hospital?"

"No, neither had any of our sisters. Nurses who are trained here will naturally be, in future, particularly eligible for the post of sister at an Ophthalmic Hospital, after they have had their general training. Hitherto we have been absolutely obliged to have sisters without special training."

### The Features of the Nursing.

"Speaking of special training, I shall be glad if you will tell me the chief features of the nursing here?"

"As you know all the patients have bad eyes in one way or another, and, of course, the primary object in view is to cure them. The nurses are taught to look after cataract cases. They attend operations; they feed the patients, learn the best way of bathing their eyes, how to put in drops, and apply lotions and instruments. There is also a good deal of tying-up and bandaging to do."

"I suppose the patients are very glad when anyone is able to read to them?"

"Yes, but the nurses have no time for that. Ladies are good enough to come and read and play to them. They sometimes get very depressed sitting in their day rooms, and thoroughly appreciate the efforts of those who try to alleviate their depression."

"One feature, continued the lady superintendent, is that the patients do not, as a rule, remain longer than three weeks. Many cases are cured in a much shorter period. In fact, new cases are admitted every day."

### General Cases.

"If a patient falls ill of any other disease, what happens?"

"Unless it is infectious we nurse such cases, and we make one exception in regard to infectious diseases. Patients who develop measles here are not sent away. There is no hospital to which they can be transferred. Sometimes we get cases of pneumonia. In these and other general cases the training of the sisters renders them equal to any emergency, and we also take opportunities like these of giving the probationers an insight into general work."

"Do you consider that, so far as a probationer is concerned, the work is lighter than in a general hospital?"

"Yes, I do; and even for staff nurses and sisters the routine work is not so hard.

### The Nurses' Quarters.

"Have you had more applications for probationerships since you extended the period of training?"

"As soon as we arrived here and got all the modern appliances which make this the best as well as the biggest eye hospital in the world, the applications began to increase. The immediate result of extending the training to two years was that we received applications from girls of better education anxious to enter as probationers. The nurses' quarters here are very comfortable."

"Yes, I saw them before they were occupied. You perhaps prefer the nurses being housed on a floor above the hospital, instead of in a separate home?"

"It is better with a small staff, and especially as the quarters include everything that could be provided in a home. Every nurse has a nice bedroom to herself; the staff nurses and probationers have a sitting-room of their own, and the sisters have one to themselves. Our nurses, on account of their youth, want more oversight than those in a general hospital, and it is an advantage to be able to look after each individually."

### The Most Essential Qualities.

"Finally I should like to know what, in your judgment, are the most essential qualities for success in nursing patients suffering from bad eyes."

"An eye nurse must be very gentle. She also needs to be very observant, because it takes so little to make an eye go wrong. But practically an eye nurse should have the same qualities which are indispensable to success in a general nurse, and which certainly include obedience, punctuality, and patience. Nothing can be done without punctuality; and here from the little babies to the old people, some of whom are very stupid as well as very blind, the utmost patience has to be exercised with the patients. Our probationers have so much to do with the nursing of children that, in effect, they pass through a considerable portion of the training given in a children's hospital."

### The State Registration of Nurses.

### MATRONS IN OPPOSITION.

A fortnight ago we published a manifesto against the State registration of trained nurses. It has now been signed by the following representatives of important nurse-training schools, as well as by a number of official personages, including upwards of 50 members of the medical profession, connected with the leading hospitals.

### Matrons of Hospitals in London.

Eva C. E. Lückes: matron, London Hospital. Katherine Monk: sister matron, King's College Hospital. Frances Elizabeth Spencer: superintendent of nurses, Edinburgh Royal Infirmary. G. M. Thorold: superintendent of nurses, Middlesex Hospital. Florence Smedley: matron, St. George's Hospital. Elizabeth M. Medill: matron, St. Mary's Hospital. Harriette E. G. Hamilton: matron, St. Thomas's Hospital. Dora Finch: matron, University College Hospital. Mabel H. Cave: matron, Westminster Hospital. Isabel C. Bennett: matron, Metropolitan Hospital. S. E. Bland: matron, Poplar Hospital. B. Marian Cooke: matron, Royal London Ophthalmic Hospital. Alice Mary Hall: matron, Seamen's Hospital, Greenwich. Louisa Halliday: matron, Royal Waterloo Hospital for Women and Children. Frances Hole: matron, National Orthopædic Hospital. Elizabeth Keith: matron, Hospital for Paralysis and Epilepsy. C. Graham Knight: matron, Royal Victoria and Albert Dock Hospital. Gertrude Knott: matron, British Lying-in Hospital. Amy F. Longbottom: matron, Westminster Training School and Home for Nurses. Kate E. Norman: sister matron, Royal Eye Hospital, Southwark. Gertrude Payne: matron, Hospital for Sick Children, Great Ormond Street. Fanny M. Phillips: matron, Grosvenor Hospital for Women. E. M. Phillips: matron, Hostel of St. Luke. Ada M. Reed: sister in charge, Private Nursing Home, Great Ormond Street. E. M. Shotter: matron, Cancer Hospital. Eleanor C. Vernet: matron, National Hospital for the Paralysed and Epileptic. J. Watson: matron, Victoria Hospital for Children, Chelsea. Elizabeth Dodds: matron, Bethnal Green Infirmary. H. E. Armstrong Dixon: matron, Greenwich Infirmary. Jane E. Styring: matron, Paddington Infirmary. Grace A. Ramsden: matron, St. Marylebone Infirmary. Elizabeth Vincent: late matron, St. Marylebone Infirmary. Ellen Jean Moir: matron, St. Pancras Infirmary. E. W. Mowat, matron: Whitechapel Infirmary. Helena Gooding: matron, Woolwich Infirmary.

### Matrons of Provincial Hospitals.

A. C. Gibson: matron, Birmingham Infirmary. Letitia S. Clark: lady superintendent, Newcastle-on-Tyne Infirmary. Emily S. Nowers: matron, North Riding Infirmary. Constance F. E. Algar: matron, Isolation Hospital, Orsett, Essex. Annie Beatrix Baillie: matron, Royal Infirmary, Bristol. A. B. Barter: matron, Children's Infirmary, Liverpool. Alice Blomfield: matron, East End Mothers' Home, Commercial Road. B. C. Bridge: late matron, Norfolk and Norwich Hospital.

Katherine Bulteel: matron, Taunton and Somerset Hospital. Margaret Cameron: matron, Brooklyn Institute, Anerley. Frances E. Court: matron, Victoria Home, Margate. Elizabeth M. Crawford: late matron, Hamilton Hospital, Bermuda. Margaret Cummings: matron, Cumberland Infirmary, Carlisle. Marianne E. Dann: sister in charge, Cottage Hospital, Welwyn. Jane S. Downes: nurse matron, Grove Hospital, Leighton Buzzard. J. M. Duff: matron, Royal Infirmary, Dundee. Susan Dunham: matron, Epsom Cottage Hospital. Alice Maud Elliot: matron, London Hospital Convalescent Home, Tankerton. Annie Croft Glover: matron, David Lewis Northern Hospital, Liverpool. Annie J. Glover: matron, Royal Albert Hospital, Devonport. Anna A. Gwyn: matron, Royal Hants County Hospital. Janet Stirling Hamilton: matron, Royal Hospital for Incurables, Putney. Kate E. Jones: matron, Deal and Walmer Victoria Hospital. Marianne Hannay: matron, Royal Sea Bathing Hospital. M. Herbert: matron, Worcester General Infirmary. Fanny Jarvis: matron, Westminster Nurses' Home, Eastbourne. Edith Jordan: matron, St. Catherine's Convalescent Home, Penn, Staffs. Emily Kay: matron, Isolation Hospital, Weymouth. Sarah Lambert: matron, Cottage Hospital, Cromer. Mary W. Lea: matron, Cottage Hospital, High Wycombe. Emily Lowe: late matron, Bagulay Sanatorium, Timperley. M. W. McDouall: matron, Rous Memorial Hospital, Newmarket. Annie Michie: lady superintendent, City and County of Worcester Nursing Institution. A. M. Monk: matron, Free Eye Hospital, Southampton. Ada M. Moore: matron, Eccles and Patricroft Hospital. Margaret Morgan: matron, Addenbrooke's Hospital, Cambridge. Marian Neepe: lady superintendent, General Hospital, Northampton. Helen Neve: matron, Haslemere Cottage Hospital. Evelyn Newman: matron, Boston Hospital. Florence Osborne: matron, Hospital St. Cross, Rugby. E. W. Paterson: lady superintendent, Leith Hospital. Katherine Rapson: matron, Warneford Hospital, Leamington. Margaret Ramsay: matron, Princess Alice Memorial Hospital, Eastbourne. M. E. Ray: matron, Lincoln County Hospital. Lucy Short: matron, Cottage Hospital, Cheshunt. Julia Simpson: matron, Hospital for Sick Children, Glasgow. Alice A. Smith: matron, Hertford General Infirmary. Margaret E. Sparshott: matron, Derbyshire Royal Infirmary. Ernesta Sproule: matron, Royal Southern Hospital, Liverpool. Margaret Stuart: lady superintendent, Workhouse Hospital, Liverpool. Anna Tillyard: matron, Medical Mission Hospital. Annie Thomas: nursing instructress, Watford Union Infirmary. J. Thomas: matron, Edinburgh City Hospital for Infectious Diseases. Ann M. Tubby: matron, Cottage Hospital, Trowbridge. Lucy Wamsley: matron, Herman de Stern Convalescent Home, Felixstowe. C. M. Wilson: matron, Passmore Edwards' District Cottage Hospital, Tilbury. Agnes J. Watt: matron, Radcliffe Infirmary, Oxford. Madeline Whitehouse: matron, Norfolk and Norwich Eye Infirmary. Elizabeth Yeats: matron, General Infirmary, Gloucester. Alice Young: matron, Cottage Hospital, Woburn.

### Presentations.

Thornhill District Nursing Association.—Miss Eleanor G. Turnell, Queen's Nurse for Thornhill and district, has been presented with a gold bangle and purse of sovereigns on the occasion of her leaving the district.

### The Nurses' Dining-hall at Guy's Hospital.

### MISS CHAPLAIN'S PICTURE.

Through the generosity of Mr. Raphael, the dining-hall in the nurses' new home at Guy's Hospital has just been enriched by a large wall-painting, the work of Miss Florence E. Chaplain. The design, which occupies the upper part of one end of the hall, is a reproduction of The Procession of the Months, executed some three years ago in water-colour by Miss Chaplain, for which she received the annual prize of £40 at the Royal Academy Schools. The Months, twelve life-size female figures, have each a symbolical meaning, and the colour-scheme, dark and sombre at the two extremes of the canvas and brightening towards the centre until it culminates in the glory of summer, fitly embodies the poetical conception of the changing seasons. Beginning on the left, January is depicted wrapped in a cloak of ice, while the distant sun, appearing dimly above the horizon, lights up an almond tree just bursting into blossom. The feet of February are tied, typifying the frost-bound condition of the earth, and she holds in her hand the first snowdrop of the year. The faint purple, discernible in the hedges in the early part of the year, has been used by the artist in the colouring of the clinging garment wrapped round the shivering form of March; and April, in more cheerful garb, is standing in a bed of violets. A Cupid is twice introduced, first with a swallow's wing, offering a rose to May, and again as the "little Next Year," asleep in the arms of November, who holds him out towards December, only to be repelled by her frosty breath. The sun, at this end of the painting, is going down behind a blue hill, and a single star lights the evening sky. The middle of the canvas glows with the tints of summer, and the months have paused in their march, while a rich profusion of flowers intensifies the effect of noonday splendour. One of the most graceful of the figures, representing September, carries a lyre, to which she sings as she goes on her way, and all through the picture flows the stream of life, in which July is bathing her feet. The delicate hues of grey and blue rain-clouds, used in the garments of the months representing the springtime, are very successfully introduced, and indeed the design is so full of suggestions that it well repays careful study. Altogether, it is a picture upon the execution of which the artist must be warmly congratulated, while the addition of such a feast of colour to their dining-hall cannot fail to give an abiding pleasure to the nursing staff.

### Novelties for Nurses.

By Our Shopping Correspondent.

### EUCINIA CREAM.

This preparation, introduced by Messrs. Sandbrook and Co., is one which I can confidently recommend to nurses, to whom it should prove very acceptable. Nurses usually find a difficulty in maintaining smooth hands, and the use of most creams and ointments is attended with various disadvantages. By the use of Eucinia cream these disadvantages are obviated. It is not necessary to wash the hands after use, as the cream appears to be so absorbed by the skin as to leave no sticky or greasy substance on the surface after the cream is rubbed well in, whilst the hands are rendered soft and comfortable by its use. I cannot urge that this is an economical preparation, as its somewhat liquid form tends to a free application, whilst its pleasant qualities induce too frequent use. It is certainly the most agreeable cream I have had in my possession.

### Everybody's Opinion.

### AN ERRONEOUS ASSUMPTION.

"Asylum Nurse" writes: May I say with reference to your remarks under this head that none of the nurses who were dismissed from Tooting Bec Asylum in connection with the "ragging" case were members of the Asylum Workers' Association. Your definition of them is, doubtless, perfectly correct.

### MIDWIFERY TRAINING FEES.

"A Trained Nurse" writes: Seeing the scheme suggested at the Mansion House meeting, may I say that a School of Midwifery is urgently needed where trained nurses could be received at a lower fee that the ordinary pupils, who have probably never been inside a hospital. There are many trained nurses who would be glad to avail themselves of midwifery training if the fees were reduced to them. Surely a trained nurse is much more useful and quicker in every way than an untrained person. At present I believe that there is not a maternity hospital which reduces its fees to trained nurses.

[Several of the larger maternity hospitals already reduce their fees to trained nurses.—Ed. The Hospital.]

### SIR WILLIAM BENNETT ON PRIVATE NURSING.

"A Frequent Contributor" writes: May I say, in answer to "Matron's" assertion that I am "laying down the law on a branch of nursing which I know nothing about," that I emphatically deny that there can be one law of decency for private nurses and another for those working in hospitals? Both are women, and the question raised by Sir William Bennett's lecture applies equally to both. It is, should a nurse be required or consent to perform services for male patients (except in cases of emergency), which the elementary instincts of decency forbid? I am of opinion that there are limits to the services of a nurse to a male patient, and that where these limits must be overstepped a male nurse should be provided. "Matron," claiming to speak for "the majority of the best and most experienced private nurses," disagrees with me. Still I feel convinced that private nurses as well as hospital nurses recognised these limitations, and, feeling that their profession is lowered and their womanhood degraded by those who set them at defiance, view with disgust the advertisements of women who vaunt their skill with the male catheter, even if these nurses still retain "the gentlemanly respect" (*sic*) (whatever that may be) of their patients.

### PORTRAITS AS PRESENTATIONS.

"Delta" writes:—The correspondence about portraits as presentations is most interesting to me, a nurse, and I think the idea a splendid one. It is extremely difficult in hospital life to choose a present which shall be of permanent value to the recipient, but with a portrait this can never be the case. And a replica, placed in a boardroom or nurses' recreation-room, would act as a stimulant to nurses to deserve a similar honour. It is encouraging to feel that The Hospital so highly approves of the suggestion, showing as it does its prompt co-operation by already negotiating with a well-known lady miniature painter. The prices quoted seem most moderate for first-class work. The right kind of matron or other hospital official is always greatly esteemed, and subordinates value even a photograph of such a person more than lay people can perhaps imagine. The shape of the features matters really very little, because it is always character that makes the countenance. Most of our best matrons and sisters are obviously noble women, and this shows plainly in their faces. Many a weary young nurse gains courage and strength from the picture of a firm yet tender and mature womanly face looking out from under a stiff white cap. And this spirit of almost veneration for such a person embodies the feeling of enthusiasm which makes every loyal heart wish to preserve the countenances of these leaders and succourers of men and women, and to express permanent honour to whom honour is due in our hospitals.

[We are glad to find that this idea seems to meet with favour.—Ed. The Hospital.]

### Appointments.

Devonshire Hospital, Buxton, Derbyshire.—Miss S. A. Hyland has been appointed matron. She was trained at Guy's Hospital, London, where she has since been ward sister, night sister, and assistant matron. She has also been matron of Irene Concentration Camp Hospital, South Africa.

General Hospital, Mandalay, Burmah.—Miss Sarah Green has been appointed matron. She was trained at the Great Northern Central Hospital, London, where she was afterwards theatre sister and night sister. She has since served for several years on plague duty in India, and received the Order of St. John of Jerusalem for her services.

South Rotherham, Handsworth, and Kiverton Park District Isolation Hospital.—Miss M. K. Phillips has been appointed matron. She was trained at Sir Patrick Dun's Hospital, Dublin, and has since been assistant matron at the Borough Hospital, Wolverhampton; matron of the Wellington Quay Joint Hospital, Newcastle-on-Tyne; and matron of the City Hospital, Wakefield.

Victoria Cottage Hospital and Dispensary, Abergavenny.—Miss S. Caroline Thorp has been appointed nurse-matron. She was trained at Crumpsall Infirmary, Manchester, and has since been matron of the Cottage Hospital, Blandford, and nursing superintendent of Friedenheim Hospital, Swiss Cottage, London.

### Where to Go.

Grand Miscellaneous Concert., Stafford House, Tuesday, April 19, at 3 p.m. in aid of the funds of the Hospital of St. John and St. Elizabeth, St. John's Wood.

### TRAVEL NOTES AND QUERIES.

By Our Travel Correspondent.

Wensley Dale (Yungfrau).—The farm at which I stayed was "Whingill," near Kirkby Stephen; landlady's name, I think, Richardson. It is really in Westmorland, but very near to Hawes, which is fine. I do not remember what we paid for our board; my impression is that it was moderate. Sanitation far from modern, but it was quite healthy, Thence I went to Barnard Castle, which I preferred. It is a town, but in three minutes you are in the woods. Send for my article on the place published in The Hospital, August 5th, 1899. Near by is Greta Bridge, also a charming place to stay at. You would, I think, be enchanted with it, and could probably be met as to terms by the landlady of the Morritt Arms (the only inn).

Cornwall, Devonshire, Surrey, and Warwick (T. H. W.).—Your questions are rather comprehensive, but I think you may consider that the cost of living does not vary greatly; further than that I can hardly go, as you give me no idea of what you wish to pay. If you will be a little more definite and give me particulars I shall be very pleased to try and help. The voyage from Penzance to the Scilly Isles costs 7s. first single, 5s. second single, and 10s. 6d. and 7s. 6d. return. The boats run three times a week, weather permitting, in the summer. I do not know how long the voyage lasts; indeed it varies considerably—I think about three hours is the usual time from Penzance to St. Mary's. You must always be prepared to remain the night if the weather should be uncertain.

Etaples for a Few Weeks (M. J. W.).—There is very little choice in accommodation at Etaples, consequently it will not be very cheap. Where there is no competition prices range high. It is a small place, and to my mind not interesting. There is no English service. It is a fishing village. Write for terms (I do not know them) to Hôtel Lion d'Or and Hôtel Chemin de Fer. If you have no particular reason for staying at Etaples, I rather think you would prefer Montreuil-sur-Mer, about seven or eight miles west of Etaples—Hôtel Renard d'Or and Hôtel Cornet d'Or.

### Echoes from the Outside World.

### Movements of Royalty.

On Thursday the King left London for a private visit to the Earl and Countess of Derby at Knowsley. At Edgehill the Royal saloon in which his Majesty travelled was detached from the train and taken on to Huyton, where the King was met by Lord Derby. At Knowsley the villagers, who had assembled in large numbers, raised hearty cheers for his Majesty, who bowed his acknowledgments. The King concluded his visit on Saturday afternoon, an immense crowd witnessing his departure from Fazakerley Station, and arrived in London shortly before nine o'clock. On Monday afternoon, after the King had held a Council at Buckingham Palace, both his Majesty and the Queen left Charing Cross Station for Denmark, where they are to take part in the celebration of King Christian's eighty-sixth birthday. The festivities are always of a family character, and among the company at Copenhagen, in addition to the King and Queen, will be the Duke and Duchess of Cumberland, Prince and Princess Charles of Denmark, Prince Charles of Sweden and Norway, the Grand Duke of Mecklenburg-Schwerin, and the Hereditary Grand Duke and Duchess of Baden.

### The Queen at the Alexandra Trust Dining-rooms.

On the morning of Thursday last week Queen Alexandra paid a surprise visit to the Alexandra Trust Dining-rooms in the City Road. The Royal party, who drove to the City Road in plain carriages without escort, no one but Sir Thomas Lipton being informed of the intended visit, arrived at 12.40, and the Queen herself asked for tickets for eight dinners at 4½d., tendering half-a-crown in payment. The young lady clerk, unaware of her Majesty's identity, pointed out that the money was 6d. short, and, to the Queen's amusement, did not execute the order until Lord de Grey came forward and gravely handed over the balance. The Queen then made a tour of the large rooms, and her identity becoming known she was received everywhere with manifestations of pleasure. Her Majesty, seeing two little girls much distressed, asked the matter, and learning that they had lost their dinner tickets at once supplied them with the necessary cash to make good the loss. Subsequently, the Queen and her party partook, in the manager's little room, of the ordinary dinner, served in the ordinary way. All the party afterwards drank coffee out of thick cups. The Queen, who made a second appearance in the public dining-room amid enthusiasm, expressed her great satisfaction with the whole of the arrangements of the establishment, and on leaving was loudly cheered.

### The Queen and Working-class Dwellings.

The Queen has sent a letter to the Housing of the Working Classes Committee of the London Council expressing her satisfaction that the addition of cupboards to the houses on the Ann Street Estate, Poplar, has been such a success. She intimated in the communication her conviction that the more this addition is enforced, the more it will be appreciated by the tenants, and she signified that it affords her great pleasure to think that it was owing to her suggestion that extra cupboard accommodation was provided.

### British Women's Emigration Association.

The annual meeting of the British Women's Emigration Association was held on Friday at the Imperial Institute. Among the ladies present being Lady Knightley, of Fawsley, Lady Victoria Campbell, Mrs. Arnold-Forster, the Hon. Mrs. Joyce, President of the Council, and Miss Lefroy, Hon Secretary. Sir Gilbert Parker, M.P., who was in the chair, gave a *résumé* of the Society's work, and said that it assisted 8,000 women to settle in different parts of the Empire. During the past year its efforts had been mainly directed towards the settlement of middle-class women in Canada, where there was a keen, business-like Government, who were doing a great deal to tempt the people of Great Britain to the Dominion. The Colonial Secretary, who moved the adoption of the Report, said that they were not sending wastrels to the Colonies, but capable, effective, and courageous individuals, and by assisting in the emigration of such persons, they might claim to be doing the Colonies unmixed good. He was happy to find that the emigrants were not left to themselves when they started on their voyage across the seas. Not only were they carefully looked after here, but he understood that in Canada and elsewhere there were committees and institutions in existence to receive and befriend them. The Duke of Argyll closed the proceedings by proposing a vote of thanks to the Chairman and the voluntary officials of the Association.

### The War in the Far East.

Despatches received in St. Petersburg from Admiral Makaroff and from the military authorities at Port Arthur state that at a very early hour on Sunday morning the Japanese made a second attempt to block the entrance to the inner roadstead. Admiral Makaroff says that the attempt has failed, that the entrance remains perfectly free. Four Japanese steamers were sunk, and a Russian torpedo-boat, but it is thought that the latter can be refloated. Proclamations have been issued by the commandant of Vladivostock indicating preparations for a siege.

### The Lord Warden of the Cinque Ports.

The King has approved of the appointment of Lord Curzon, Viceroy of India, to be Lord Warden of the Cinque Ports in the room of the late Marquis of Salisbury. There are no emoluments attached to the ancient office, but it is only conferred upon persons who have rendered distinguished service to the country, and it carries with it the enjoyment of the charming residence known as Walmer Castle. The Castle itself was built by Henry VIII., but the first Warden of the Cinque Ports—Sandwich, Dover, Hythe, Romney, and Hastings—was appointed by William the Conqueror. William Pitt was Lord Warden in the latter part of the eighteenth century, and the Duke of Wellington from 1829 to 1852.

### Death of Sir Edwin Arnold.

The death of Sir Edwin Arnold was announced last week, and his remains have since been cremated, the funeral taking place on Monday. Sir Edwin had for some time been in failing health, but though nearly blind and partially paralysed he maintained his cheerfulness, and his interest in life and literature to the last. Born June 10, 1832, he was in his seventy-second year, and was thrice married, his last wife being a Japanese lady who survives him. Sir Edwin Arnold had been so long connected with the *Daily Telegraph*, of which he was editor-in-chief for many years, that few knew he started life as assistant master at King Edward's School, Birmingham, and was afterwards for a time Principal of the Government Sanscrit College at Poona, an office which he held during the Indian Mutiny. As well as the many thousands of leading articles which he contributed to the *Telegraph*, he published several independent works, of which the most important is *The Light of Asia*. He was knighted in 1888, and in addition to the Star of India, he possessed orders from Japan, Siam, Turkey and Persia.

### Notes and Queries.

**REGULATIONS.**

**The Editor is always willing to answer in this column, without any fee, all reasonable questions, as soon as possible.**

**But the following rules must be carefully observed :—**

1. **Every communication must be accompanied by the name and address of the writer.**
2. **The question must always bear upon nursing, directly or indirectly.**

**If an answer is required by letter a fee of half-a-crown must be enclosed with the note containing the inquiry.**

*Home.*

(1) 1. Will you kindly tell me where a helpless elderly woman can be received? She has a small income of about 10s. a week. 2. Please let me know the hours for out-patients at the Ophthalmic Hospital, City Road.—*Nurse H.*

1. Apply the Hon. Secretary, Woodside Home, Whetstone, N.; or to St. Peter's Sisterhood, Mortimer Road, Kilburn, N.W. 2. Write to the Secretary of the Hospital.

I have a small ailment from which I hope to recover. Can you tell me of a comfortable home, for 12s. or 14s. weekly, where I could be received, and where I could remain permanently if I did not get well? I trust I am not wrong in asking, but I heard that you would always help nurses, and I am one, retired.—*E.P.*

Probably the Woodside Home, Whetstone, N., is what you want.

*Wool-skein Truss.*

(2) Will any of your readers kindly tell me how to make and apply a wool-skein truss?

Take a skein of soft white wool, do not cut, but apply double round the waist of the child. Put one end through the *other* end and *knot* over the hernia, then bring the long end *under* the thigh, bring it *upwards* and *over* the thigh, close to groin, and knot again over the hernia to finish.

*Book upon Bacteriology.*

(3) Will you kindly tell me of a book upon Bacteriology suitable for nurses who wish to learn the first elements of this subject?—*Sister T.*

"Bacteriology of Every-day Practice," by J. O. Symes, M.D., 2s. 9d. post free.

*Nursing Section.*

(4) Nurse T. would like to know if it is possible to obtain the Nursing Section of the "Hospital" only? If so, can she order it through a newsagent, or must she send direct to the "Hospital" Office for it?

The Nursing Section of "The Hospital" is not sold separately, but nurses frequently arrange to share copies of the paper, and some send on the paper to a fellow-nurse for the cost of the postage.

*Indian Hospitals.*

(5) May I trouble you to send me a list of civil hospitals in India, and instructions how to make application for appointments in them?—*S.E.S.*

Burdett's "Hospitals and Charities" gives a list of Indian hospitals. Apply direct to the Nursing Superintendents.

Will you kindly give me information as regards the hospitals and nursing homes in India, and tell me how to apply for work?—*Sister Elsa.*

See reply to S.E.S. You might obtain work through the Up-country Nursing Association (apply to Mrs. Sheppard, 10, Chester Place, Regent's Park, N.W.), or through the Colonial Nursing Association, Imperial Institute, S.W.

**Important Nursing Textbooks.**

"The Nursing Profession: How and Where to Train." 2s. net; 2s. 4d. post free.

"A Handbook for Nurses." By. Dr. J. K. Watson. 5s. net; 5s. 4d. post free.

"Practical Guide to Surgical Bandaging and Dressings." By Wm. Johnson Smith, F.R.C.S. 2s. post free.

"The Nurses' Dictionary of Medical Terms and Nursing Treatment." By Honnor Morten. 2s. post free

"The Human Body: its Personal Hygiene and Practical Physiology." By B. P. Colton. 5s. post free.

"Art of Feeding the Invalid." (Popular Edition). 1s. 6d. post free.

"On Preparation for Operation in Private Houses." By Stanhope Bishop, F.R.C.S. 6d. post free.

### For Reading to the Sick.

EASTERTIDE.

After the Resurrection joy
The Easter light,
After the dawn has chased away
The shades of night,
After the eyes of love have seen
The Risen Lord,
After we know the blessedness
Of joy restored,
There must be closer watch to guard
The way we take,
There must be loving service done
For His dear sake,
There must be living in the light
Which Easter gives,
There must be holier life in us
Because He lives. *A. R. G.*

He said, "I am the Resurrection and the Life"; not "I shall be," but I am, even in the crisis of bereavement, even in the prospect of the Cross; not the Resurrection only, but the Life, that permanent and eternal power of which it is one result; but only one result—that men shall rise again. . . .

Thus there are two main thoughts in this revelation of Christ. It teaches us that the Resurrection and the Life in which we believe are realities which are not future only but present; it teaches us also that both lie in our union with a Person. Our faith, in the last trial to which it must be subjected, reaches out beyond the seen, and aspires to a fulness of being which transcends all experience.—*Bishop Westcott.*

The Christian doctrine of the resurrection of the body does not imply that the particles of our earthly bodies, which have dissolved in the grave and passed into various forms of natural life, will be brought together again; but that we "in our same selves shall be reclothed in a spiritual body, which we shall recognise as our own body." "It is sown a natural body; it is raised a spiritual body"—this is the Christian doctrine.—*V. Stanley.*

Let us try this Eastertide, with freshness of hope, simply to clear away, God helping us, whatever checks the free expansion of the risen life within us; whatever breaks and spoils the work of grace. We have failed, it may be, a thousand times in the years that are past; we have drifted to and fro, and hardly know whether we are any nearer the haven than we were. But it need not be so now; that is not what Christ died and rose again to win for us. We shall not be faultless in the future; but we may do better than we have done, and then better, and better still. Only let us be definite, and let us be humble; let us look right away from ourselves, right up to Him.—*Bishop Paget.*

On that happy Easter morning
All the graves their dead restore;
Father, sister, child, and mother,
Meet once more.

To that brightest of all meetings
Bring us, Jesus Christ, at last;
By Thy Cross, through death and judgment,
Holding fast.

*Rev. S. Baring-Gould.*

